# Supplementary material for: A 2D material–based transparent hydrogel with engineerable interference colours
Source: Nat Commun. 2022 Mar 8;13:1212. doi: 10.1038/s41467-021-26587-z (PMC8904793; doi:10.1038/s41467-021-26587-z)
Supplement: Supplementary file 1 — Supplementary Information [file 41467_2021_26587_MOESM1_ESM.pdf]

Supplementary information

A transparent hydrogel with engineerable interference colours

Baofu Ding[1,4], Pengyuan Zeng[1,4], Ziyang Huang[1], Lixin Dai[1], Tianshu Lan[1], Hao Xu[1], Yikun Pan[1], Yuting Luo[1], Qiangmin Yu[1], Hui-Ming Cheng[1,2,3] and Bilu Liu[1]*

[1] Shenzhen Geim Graphene Center, Tsinghua-Berkeley Shenzhen Institute and Institute of Materials Research, Tsinghua Shenzhen International Graduate School, Tsinghua University, Shenzhen 518055, China

[2] Shenyang National Laboratory for Materials Science, Institute of Metal Research, Chinese Academy of Sciences, Shenyang 110016, China

[3] Advanced Technology Institute, University of Surrey, Guildford GU 27XH, UK

[4] These authors contributed equally

Supplementary Table 1| Summary of fabrication conditions of all CTO based MB-hydrogels in this work.

Sample	Concentration ($\times 10^{-2}$ vol%) *C*	Magnetic field (mT) $\mu_0 H$	Shape	Thickness (mm) *L*	Size or Diameter (mm)
MB-hydrogel-1	2	700	Cylinder	2	**D** = 10
MB-hydrogel-2	2	700	Cylinder	10	**D** = 10
MB-hydrogel-3	2	220	Cylinder	2	**D** = 6
MB-hydrogel-4 ~ 12	2	0 ~ 800<br>Step = 100	Cylinder	2	**D** = 6
MB-hydrogel-13 ~ 24	2	160 ~ 1040<br>Step = 80	Cylinder	6	**D** = 6
MB-hydrogel-25 ~ 27	0.8	221, 397, 730	Cylinder	5	**D** = 6
MB-hydrogel-28	0.8	0 ~ 400	Cube	5	**W** = 10<br>**H** = 45
MB-hydrogel-29	12	360 for "T"<br>480 for "B"<br>600 for "S"<br>720 for "I"	Cube	1	**W** = 20<br>**H** = 45
MB-hydrogel-30	12	300 ~ 480	Cube	1	**W** = 20<br>**H** = 20
MB-hydrogel-31	2	700	Cube	10	**W** = 10<br>**H** = 10

Note: **D** represents the diameter for cylindric hydrogels. **W** and **H** represent the width and the height for cubic hydrogels.

Supplementary Table 2 Summary of birefringence (Δn) versus magnetic field for various magnetic hydrogels.

Materials	Magnetic field (T)	Birefringence (× 10^{-5})	Transmittance (for 10 mm)	Reference
CTO (0.02 vol%)	0.8	20	Transparent	This work
CTO (0.05 vol%)	0.8	40	Transparent	This work
**Disc-like material**				
Tm^{3+}-chelating bicelles	5.5	0.40	Transparent	1-3
Dy^{3+}-chelating bicelles	5.5	0.37	Transparent	1,2,4
Polymersome	20	0.3	Transparent	5
2D Nontronite	9	3.0	Transparent	6
2D Titanate	10	N/A	Transparent	7,8
**Rod-like material**				
Fibrinogen	11	2.8	Transparent	9
Pb-doped silica	10	0.10	Transparent	10
PHBV	2	1.50	Transparent	11
DPPC	5.5	0.015	Transparent	2
Agarose	3	0.030	Transparent	12
**Sphere-like material**				
γ-Fe_2O_3	1.2	20	Opaque	13

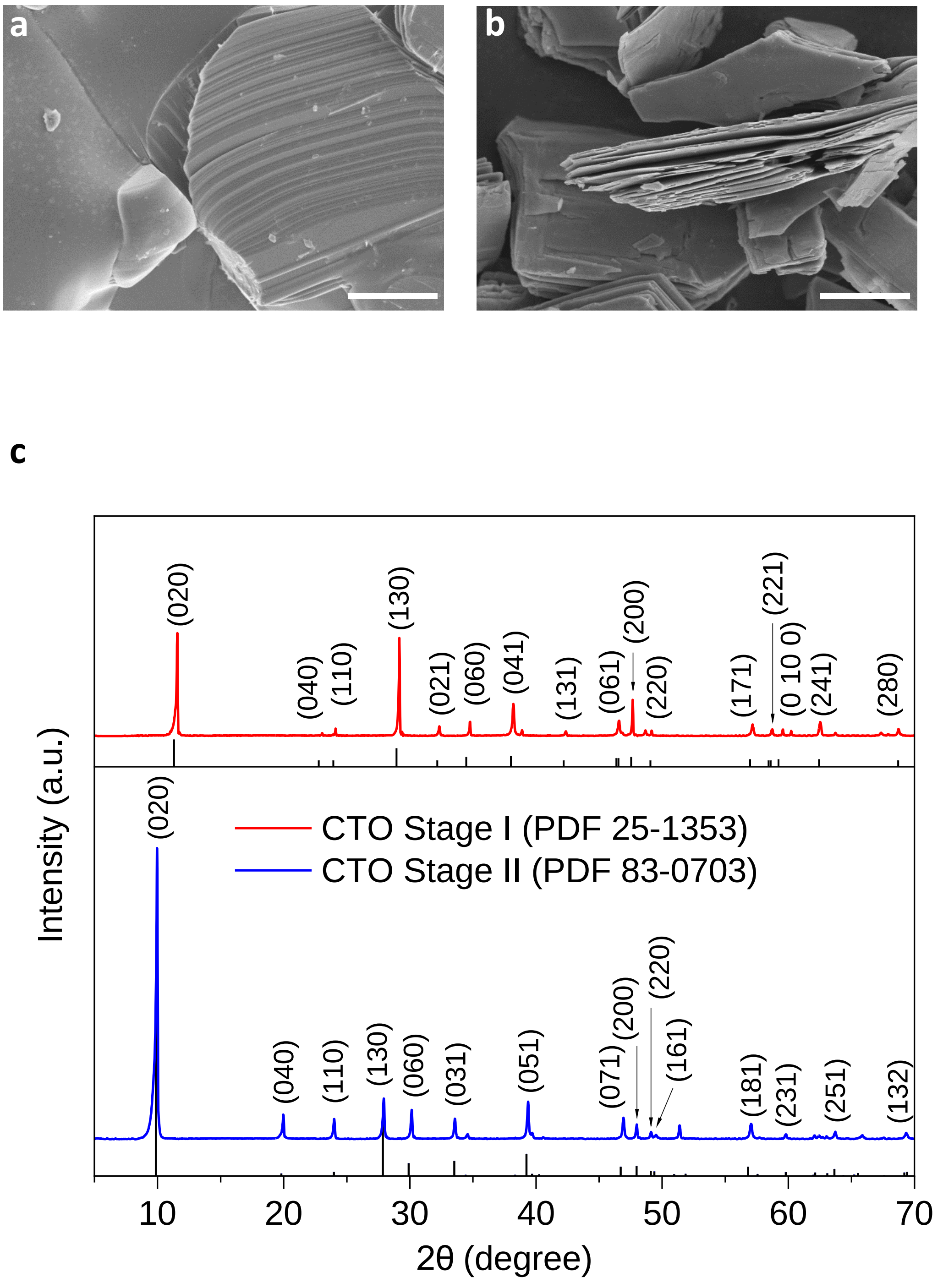

Supplementary Fig. 1 Synthesis and characterisation of the layered parent compound for 2D $Ti_{0.83}Co_{0.2}O_2^{0.47-}$ materials (CTO). **a, b,** Scanning electron microscope (SEM) images of the $K_{0.8}Ti_{1.67}Li_{0.13}Co_{0.2}O_4$ (Stage I product) (**a**) and $H_{0.93}Ti_{1.67}Co_{0.2}O_4$ (Stage II product) (**b**) with layered structures. A much-expanded interlayer spacing for $H_{0.93}Ti_{1.67}Co_{0.2}O_4$ was obtained due to the exchange between proton and alkali ions. **c,** X-ray diffraction (XRD) pattern of the Stage I product $K_{0.8}Ti_{1.67}Li_{0.13}Co_{0.2}O_4$ and the State II product $H_{0.93}Ti_{1.67}Co_{0.2}O_4$.

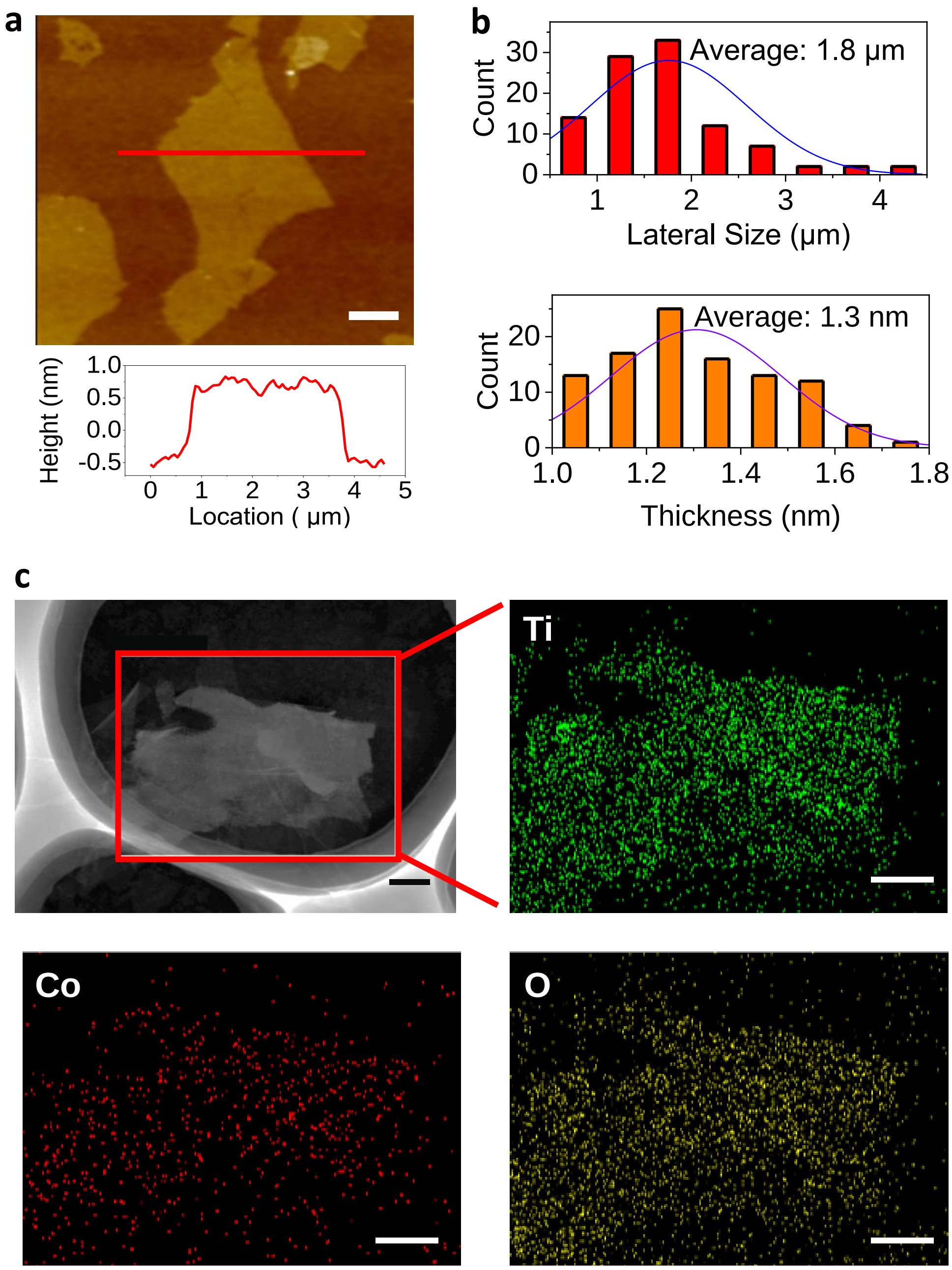

Supplementary Fig. 2 Synthesis and characterisation of the exfoliated 2D CTO materials. a, Thickness and lateral size of the 2D CTO materials characterised by atomic force microscopy (AFM). A typical AFM image of a 2D CTO sample shows a lateral size of ~ 3 μm and a thickness of 1.1 nm. Scale bar is 1 μm. **b,** Size statistics of 2D CTO materials. The logarithmic normal distribution function is used to fit the distribution. Mean lateral size and thickness of 2D CTO materials were determined to be 1.8 μm and 1.3 nm. Aspect ratio, as defined by dividing mean lateral size with mean thickness, is 1.4×10^3. The data for lateral size and thickness calculation were obtained from n = 100 independent flakes. **c,** A TEM image and energy-dispersive X-ray spectroscopy (EDX) mapping of the elemental composition of 2D CTO. The mapping results show that Co is uniformly doped into the matrix of TiO_2. Scale bar is 0.1 μm.

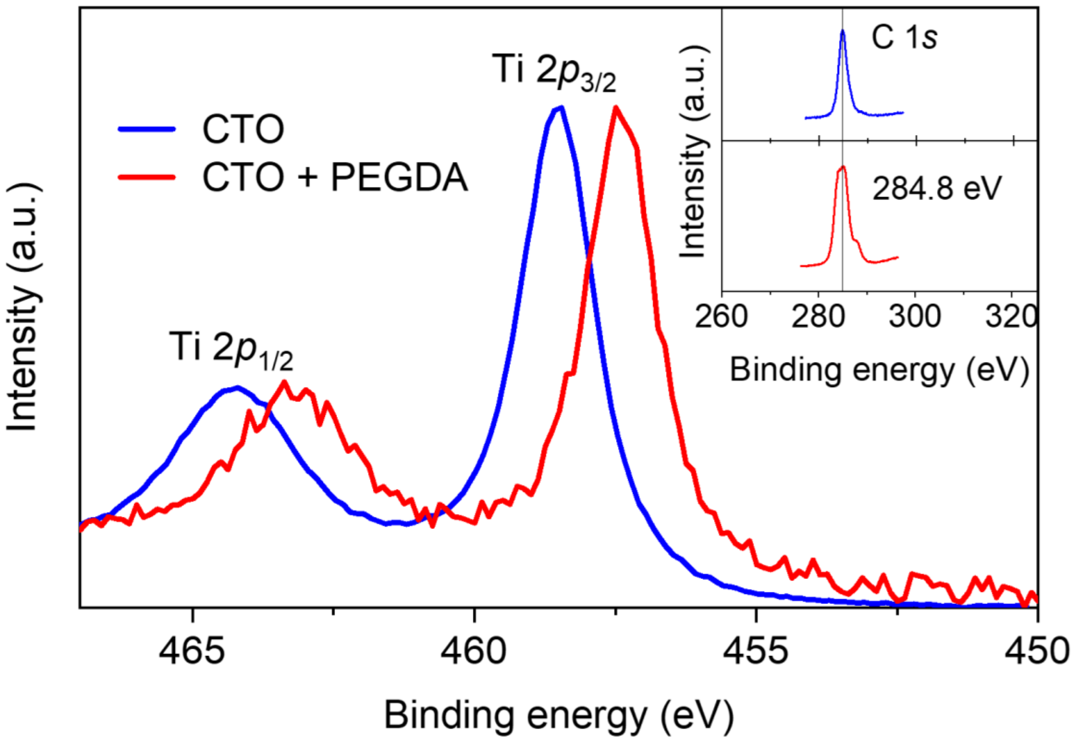

Supplementary Fig. 3 Characterization of interface interaction between 2D CTO and polymer network in hydrogel. XPS spectra of Ti 2$p_{3/2}$ of two filtered films based on pure 2D CTO flakes (blue curve) and polymer-PEGDA modified 2D CTO flakes (red curve), the Ti 2$p_{3/2}$ peaks located at 458.47 eV and 457.44 eV, respectively. Inset shows the XPS spectra of C 1*s* peaks at 284.8 eV used for calibration.

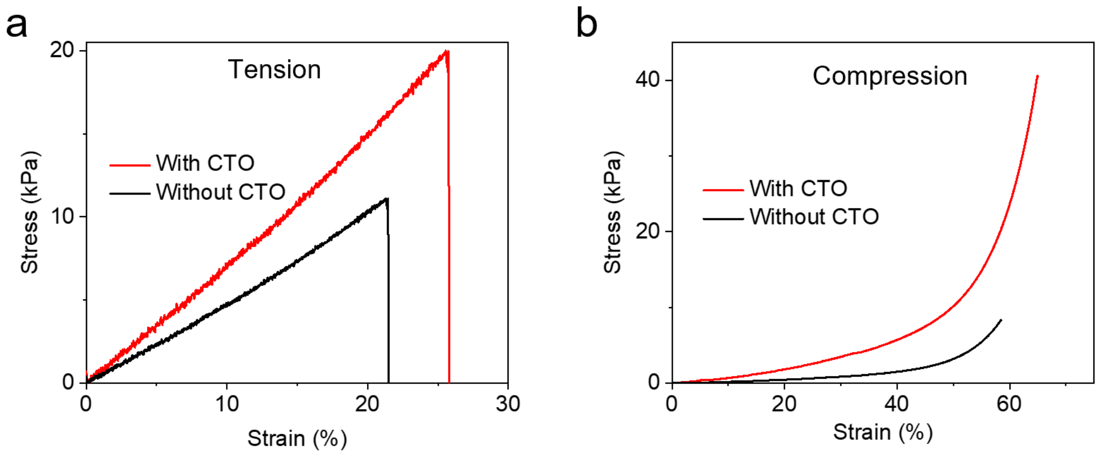

Supplementary Fig. 4 Mechanical properties of hydrogels without and with CTO. **a**, **b,** Stress-strain curves for tension (**a**) and compression (**b**).

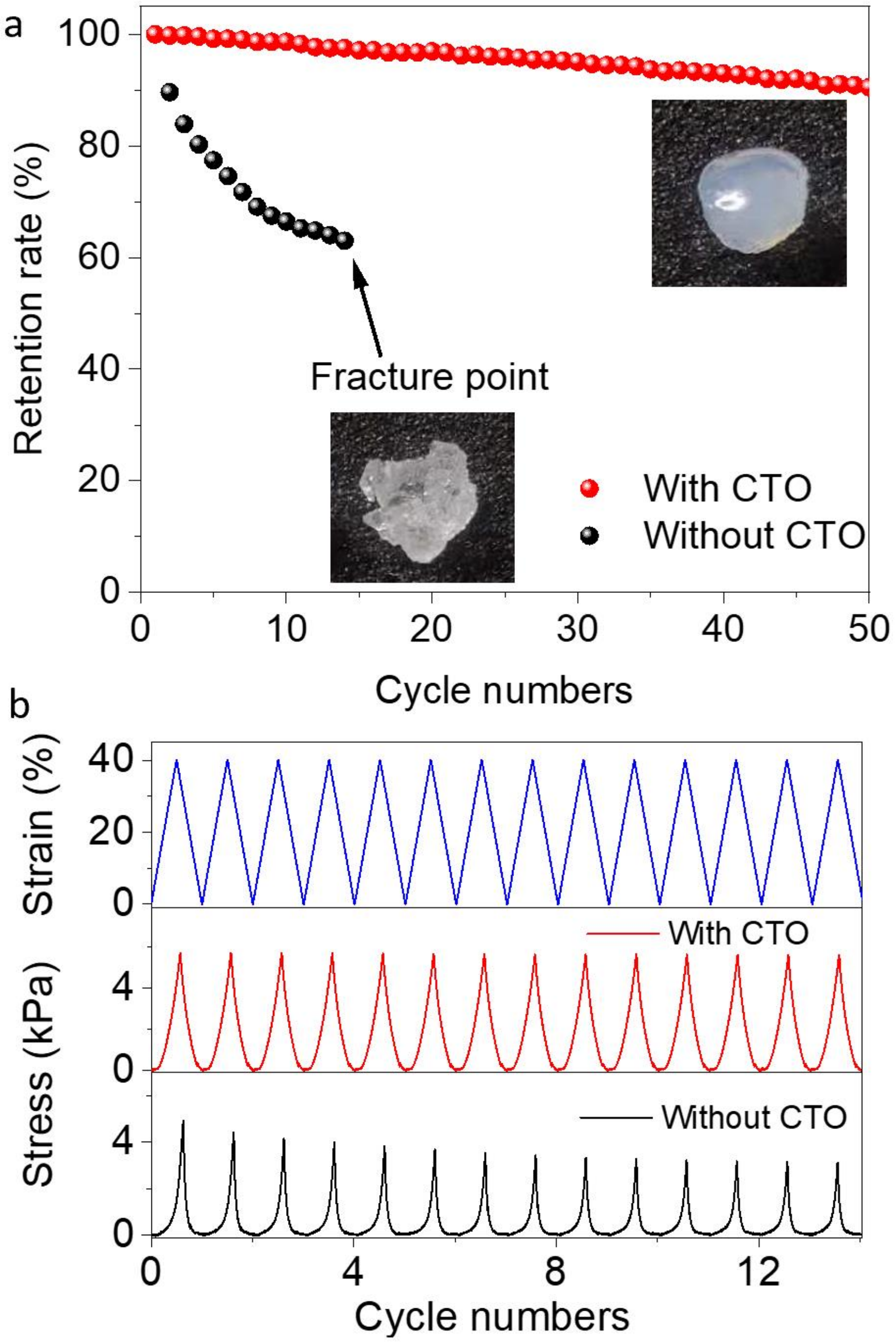

Supplementary Fig. 5 Cycling performance of hydrogel without and with CTO. **a**, Retention rates for hydrogels without CTO (black curve), with CTO (red curve). **b**, Strain (top panel), compressions of hydrogels without CTO (middle panel) and with CTO (bottom panel) for the first 14 cycling periods.

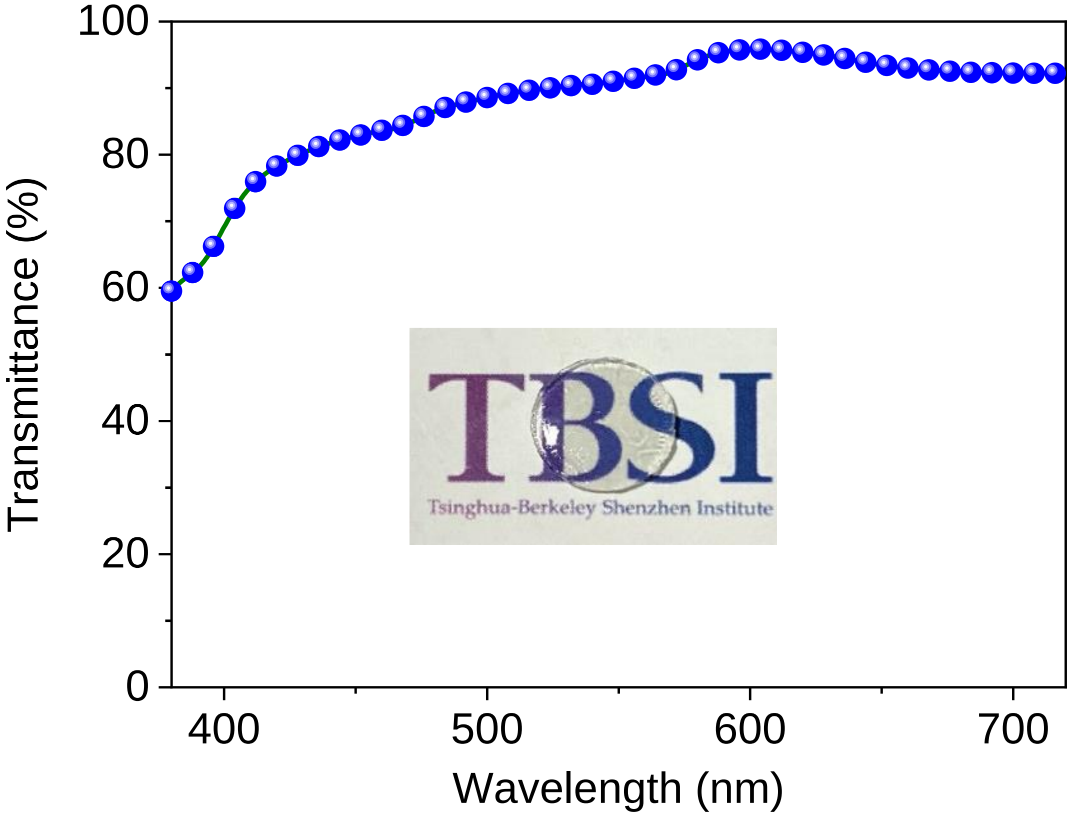

Supplementary Fig. 6 Transmittance of a typical MB-hydrogel. The incident light is non-polarized. Inset shows the MB-hydrogel-1 with a concentration 0.02 vol% of 2D CTO materials, a diameter of 10 mm and a thickness of 2 mm. The result shows that the MB-hydrogel is highly transparent in the visible range.

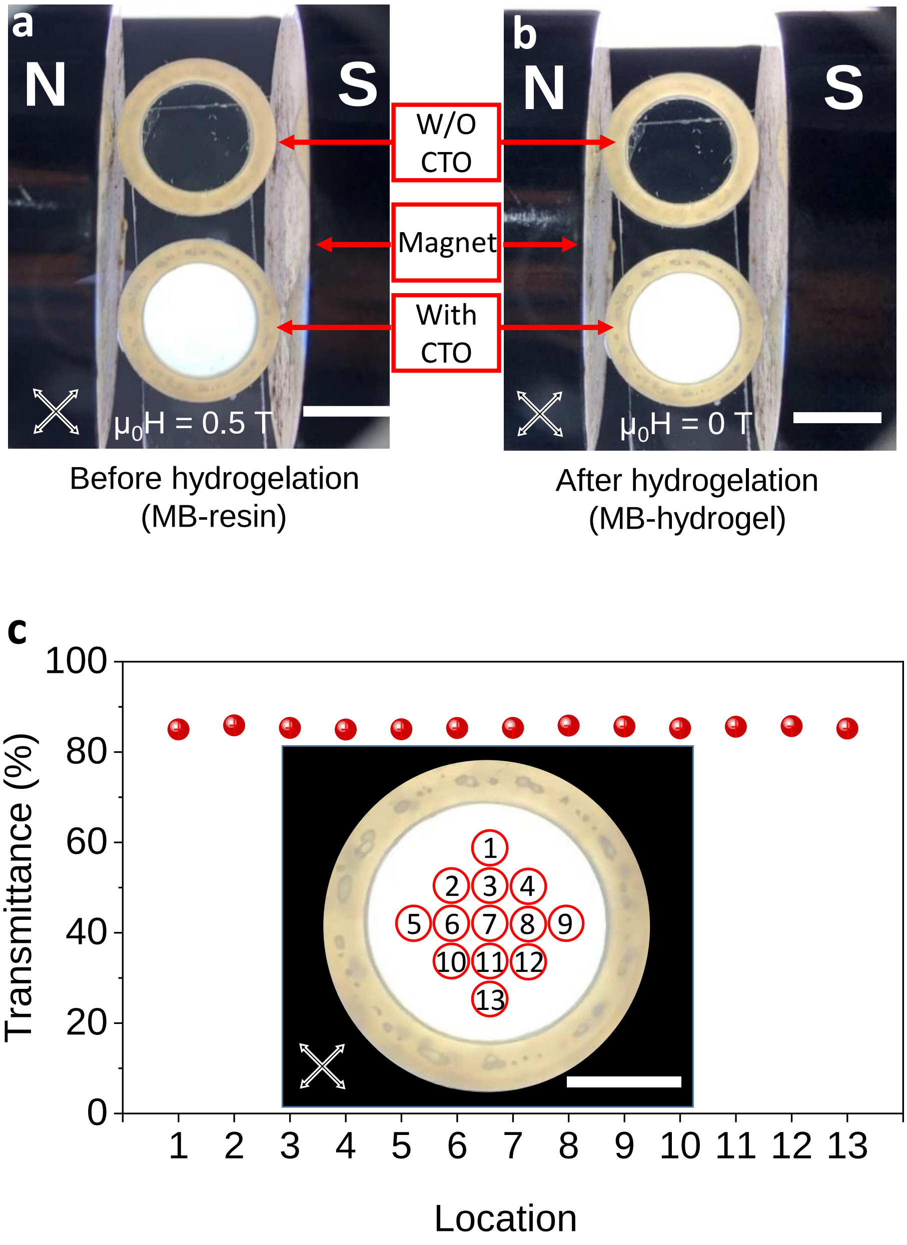

Supplementary Fig. 7 Preservation and uniformity of magnetic-field induced optical anisotropy of MB-hydrogel. a, b, Polarized images of MB-resins in the presence of magnetic field (**a**) and the corresponded MB-hydrogels in the absence of magnetic field (**b**). Dark images (top part) indicate the optical isotropy of the MB-resin/MB-hydrogel without CTO. The similar bright images (bottom part) of the MB-resin and the MB-hydrogel with 0.02 vol% 2D CTO confirm the maintenance of magnetic-field induced optical anisotropy after hydrogelation. Scale bar, 10 mm. **c,** Light transmittance of the CTO based MB-hydrogel with crossed polarizers. The variation for transmittances at 13 different spots within the 6 mm × 6 mm area are less than 1%, indicating the high uniformity in optical anisotropy by using magnetic field control. Scale bar, 5 mm.

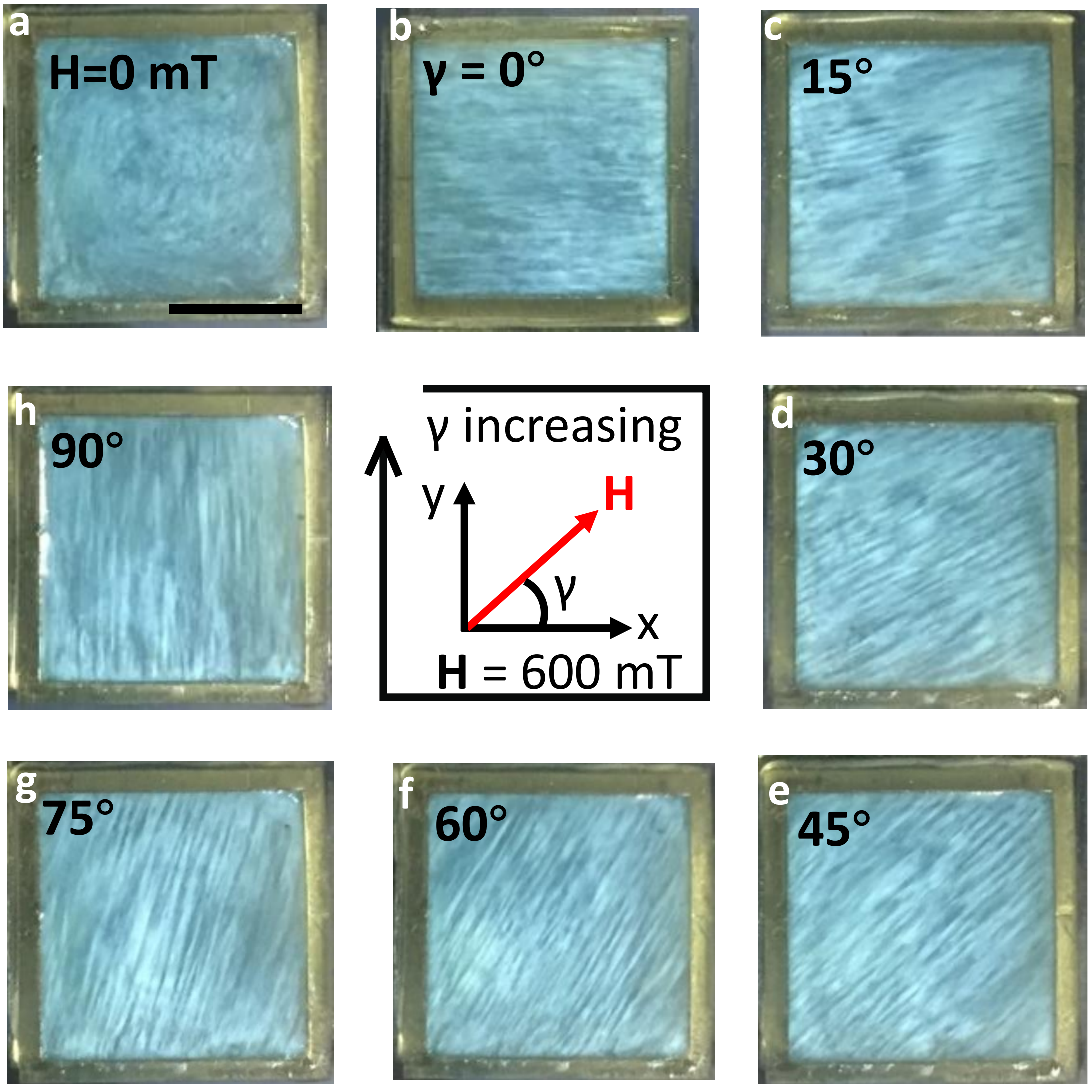

Supplementary Fig. 8 Magnetic-field induced alignment of CTO domains in the highly concentrated MB-resin. a, Snapshot of the MB-resin with the concentration of 0.2 vol% 2D CTO materials in the absence of magnetic field. **b-h,** Snapshots of the MB-resin in a rotatable magnetic field, ranging from 0° (**b**) to 90° (**h**). These photos show the parallel oriented domains of 2D CTO materials along with the external magnetic field. Scale bar: 5 mm.

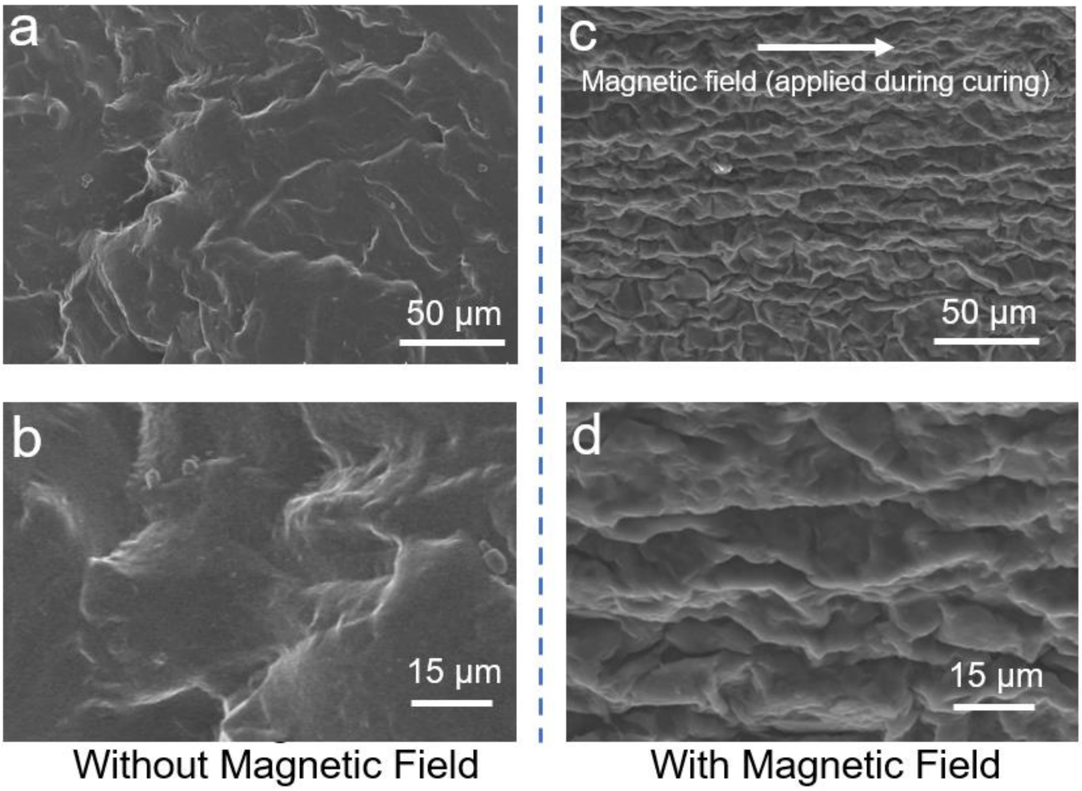

Supplementary Fig. 9 Study of ordered arrangement of CTO in MB-hydrogel made without or with magnetic field. a-d SEM images of freeze-dried MB-hydrogels cured without (**a,b**) and with (**c,d**) an external magnetic field of 1 T.

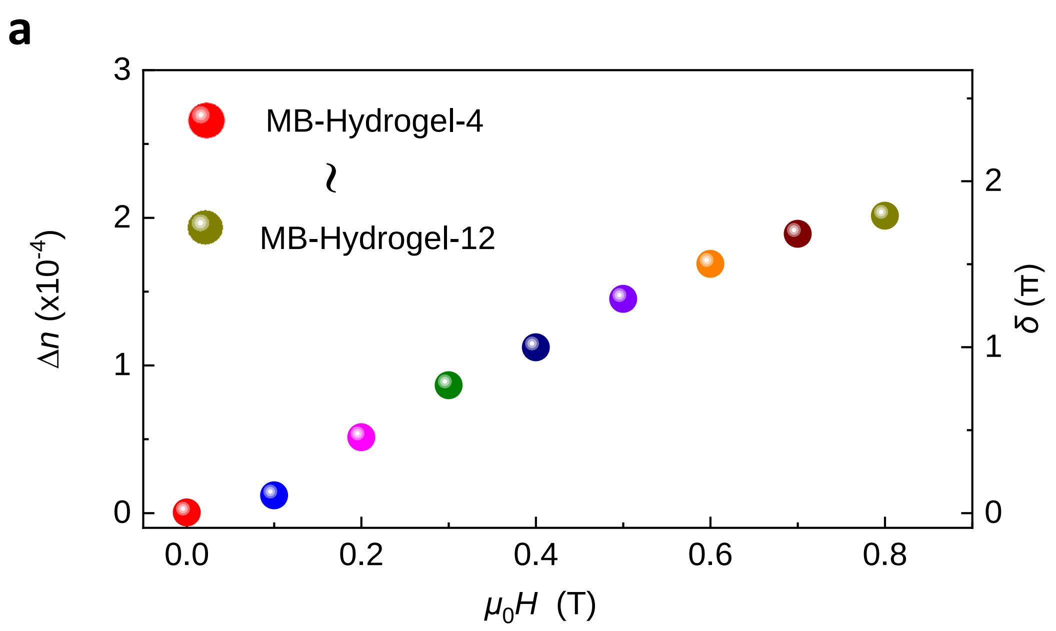

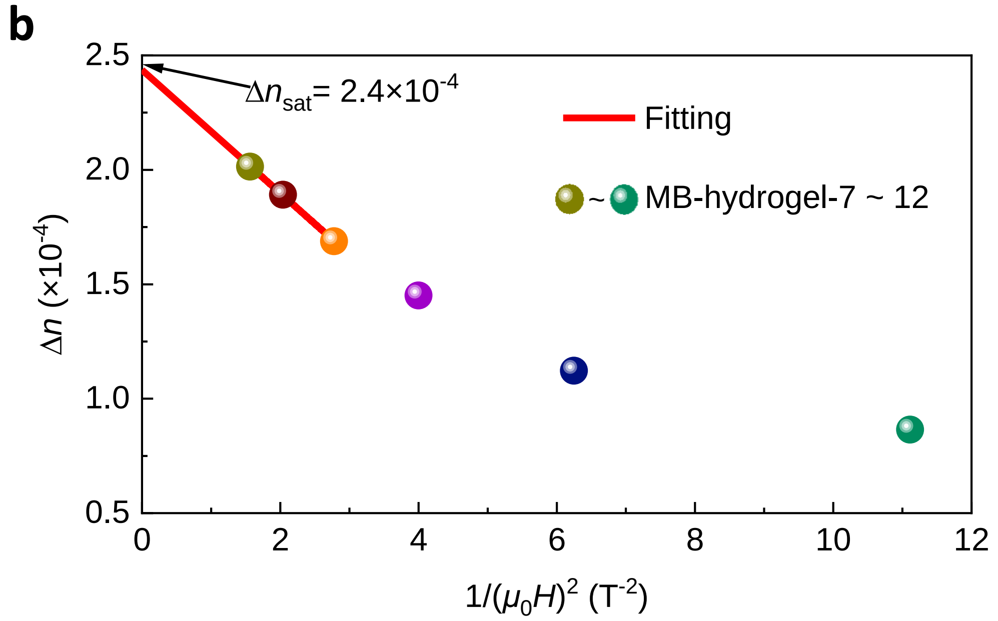

Supplementary Fig. 10 Magneto-birefringence of the MB-hydrogel. a, Field-birefringence (**H**-Δn) correspondence for the MB-hydrogels in the range of 0 ~ 800 mT. Phase retardation is calculated from Δn according to $\delta = \frac{2\pi\Delta nL}{\lambda}$, where L thickness and λ wavelength for all 9 MB-hydrogels. **b,** Plot of Δn vs $1/(\mu_0 H)^2$ to obtain the saturate birefringence value**.**

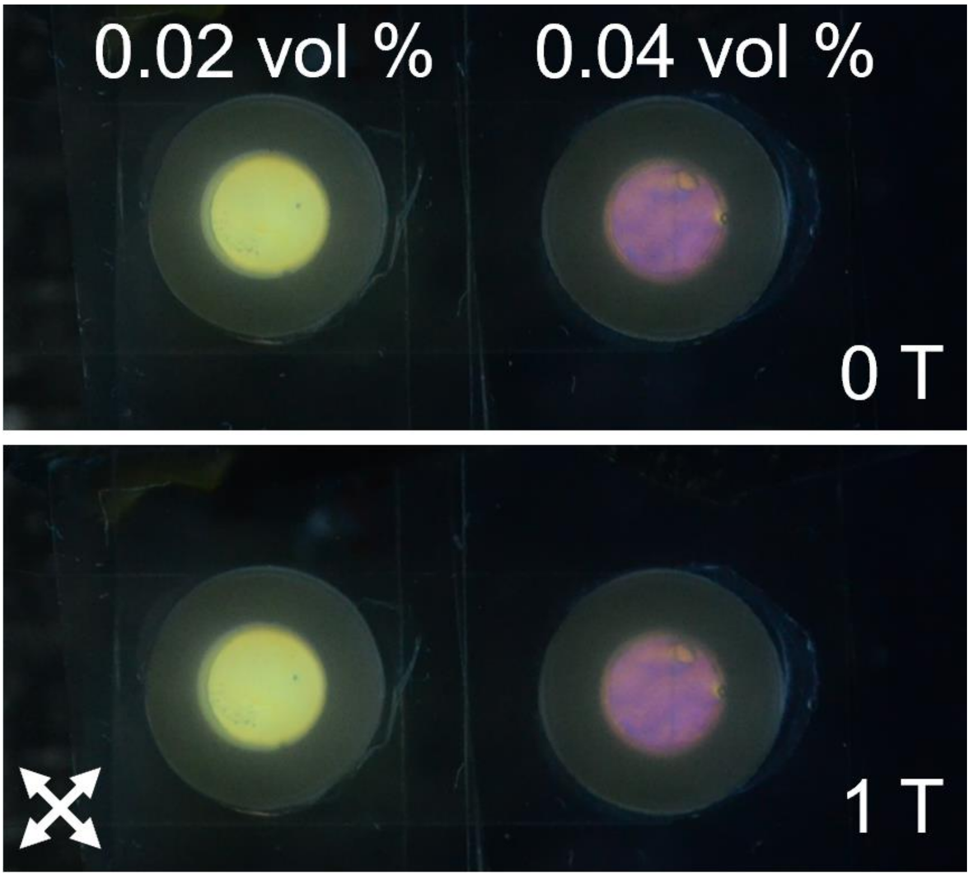

Supplementary Fig. 11 Polarized optical images of MB-hydrogels in different magnetic fields. Two already-formed MB-hydrogels with different CTO concentrations in the absence (top panel) and presence (bottom panel) of external magnetic field of 1 T. No colour change is seen, indicating once polymer is cured and hydrogel is formed, its color will be fixed and keep stable under different magnetic field.

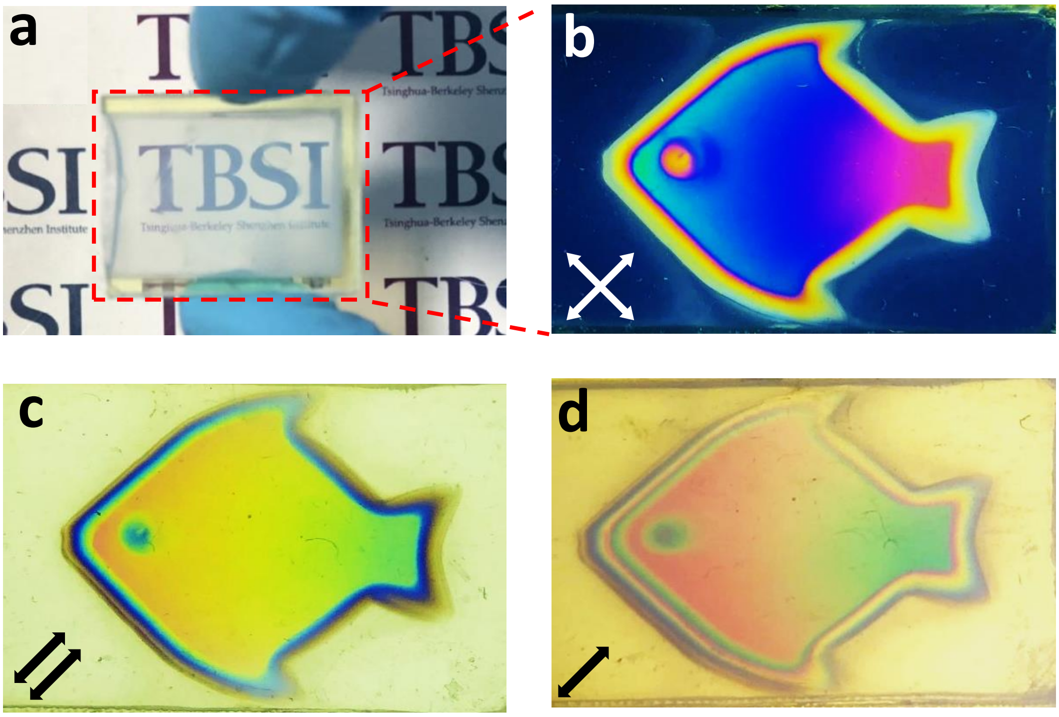

Supplementary Fig. 12 Boesemani-rainbowfish-like hydrogel under various optical backgrounds. a-d, Snapshots of MB-hydrogel-30 without any polarizer **(a),** with crossed polarizers **(b)**, with parallel polarizers **(c)**, and with a front polarizer and a back reflective mirror **(d)**.

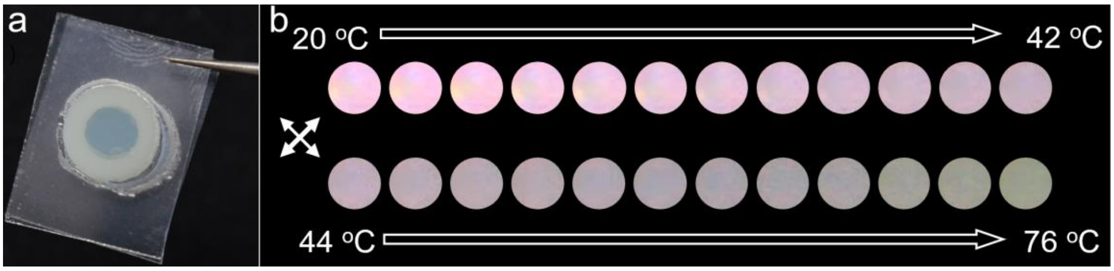

Supplementary Fig. 13 Thermochromic effect of the MB-hydrogel. a, A photo of a MB-hydrogel with transparent substrates encapsulate on both sides to avoid water loss during heating/cooling process. **b**, Colour evolution of the MB-hydrogel from pink to light green with the increase of temperature. The images were taken under two crossed polarizers as indicated.

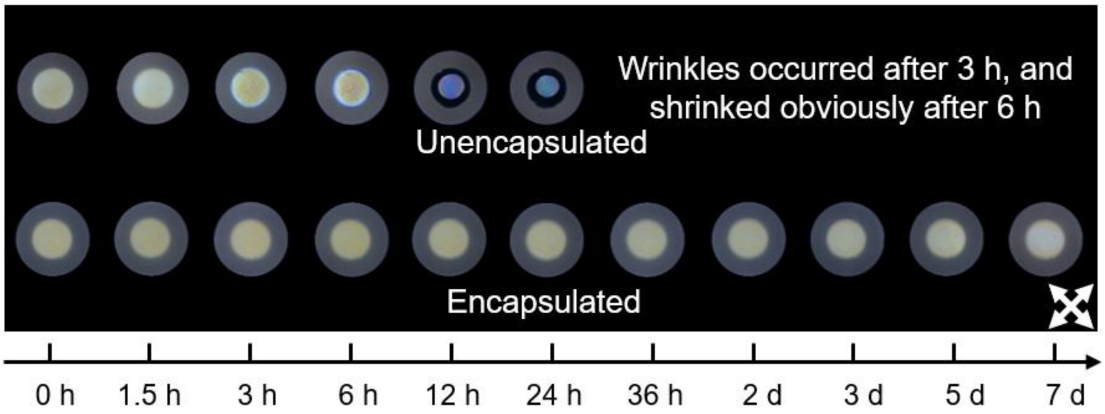

Supplementary Fig. 14 Stability of the MB-hydrogel without and with encapsulation. Bottom encapsulated hydrogel was sealed by using waterproof glue and packaging substrates.

Reference

1. Isabettini, S., Stucki, S., Massabni, S., Baumgartner, M. E., Reckey, P. Q., Kohlbrecher, J., Ishikawa, T., Windhab, E. J., Fischer, P. & Kuster, S. Development of Smart Optical Gels with Highly Magnetically Responsive Bicelles. *ACS Appl. Mat. Interfaces* **10**, 8926-8936 (2018).
2. Liebi, M., Kuster, S., Kohlbrecher, J., Ishikawa, T., Fischer, P., Walde, P. & Windhab, E. J. Magnetically Enhanced Bicelles Delivering Switchable Anisotropy in Optical Gels. *ACS Appl. Mat. Interfaces* **6**, 1100-1105 (2014).
3. Liebi, M., van Rhee, P. G., Christianen, P. C. M., Kohlbrecher, J., Fischer, P., Walde, P. & Windhab, E. J. Alignment of Bicelles Studied with High-Field Magnetic Birefringence and Small-Angle Neutron Scattering Measurements. *Langmuir* **29**, 3467-3473 (2013).
4. Tan, C., Fung, B. M. & Cho, G. Phospholipid Bicelles That Align with Their Normals Parallel to the Magnetic Field. *J. Am. Chem. Soc.* **124**, 11827-11832 (2002).
5. van Rhee, P. G., Rikken, R. S. M., Abdelmohsen, L. K. E. A., Maan, J. C., Nolte, R. J. M., van Hest, J. C. M., Christianen, P. C. M. & Wilson, D. A. Polymersome magneto-valves for reversible capture and release of nanoparticles. *Nat. Commun.* **5**, 5010 (2014).
6. Yook, S., Shams Es-haghi, S., Yildirim, A., Mutlu, Z. & Cakmak, M. Anisotropic hydrogels formed by magnetically-oriented nanoclay suspensions for wound dressings. *Soft Matter* **15**, 9733-9741 (2019).
7. Liu, M., Ishida, Y., Ebina, Y., Sasaki, T., Hikima, T., Takata, M. & Aida, T. An anisotropic hydrogel with electrostatic repulsion between cofacially aligned nanosheets. *Nature* **517**, 68 (2014).
8. Kim, Y. S., Liu, M., Ishida, Y., Ebina, Y., Osada, M., Sasaki, T., Hikima, T., Takata, M. & Aida, T. Thermoresponsive actuation enabled by permittivity switching in an electrostatically anisotropic hydrogel. *Nat. Mater.* **14**, 1002 (2015).
9. Torbet, J., Freyssinet, J. M. & Hudry-Clergeon, G. Oriented fibrin gels formed by polymerization in strong magnetic fields. *Nature* **289**, 91-93 (1981).
10. Mori, A., Kaito, T., Furukawa, H., Yamato, M. & Takahashi, K. Birefringence of silica hydrogels prepared under high magnetic fields reinvestigated. *Mater. Res. Express* **1**, 045202 (2014).
11. Stopin, A., Rossignon, A., Keshavarz, M., Ishida, Y., Christianen, P. C. M. & Bonifazi, D. Polarization of Soft Materials through Magnetic Alignment of Polymeric Organogels under Low-Field Conditions. *Chem. Mater.* **28**, 6985-6994 (2016).
12. Yamamoto, I., Saito, S., Makino, T., Yamaguchi, M. & Takamasu, T. The anisotropic

properties of magnetically ordered gel. *Sci. Technol. Adv. Mater.* **7**, 322-326 (2006).

13. Galicia, J. A., Cousin, F., Dubois, E., Sandre, O., Cabuil, V. & Perzynski, R. Static and dynamic structural probing of swollen polyacrylamide ferrogels. *Soft Matter* **5**, 2614-2624 (2009).
