# Supplementary material for: A 2D material–based transparent hydrogel with engineerable interference colours
Source: Nat Commun. 2022 Mar 8;13:1212. doi: 10.1038/s41467-021-26587-z (PMC8904793; doi:10.1038/s41467-021-26587-z)
Supplement: Supplementary file 3 — Description of Additional Supplementary Files [file 41467_2021_26587_MOESM3_ESM.pdf]

Description of Additional Supplementary Files

Supplementary Movie 1: Description

Transparency and elasticity of the as fabricated free-standing MB-hydrogel-2. The detailed parameters of the MB-hydrogel-2 are listed in Supplementary Table 1.

Supplementary Movie 2: Description

Light scattering by the MB-hydrogel-2 for different angles between scattered light and ۶-axis. ۶-axis is the direction of horizontally applied magnetic field during hydrogelation. Incident light enters from the bottom.

Supplementary Movie 3: Description

The colour evolution of the MB-resin with different concentrations of 2D CTO. CTO stands for the cobaltdoped titanium oxide materials. The video is taken in the presence of crossed polarizers. Magnetic field is tuned in the range of 0 to 700 mT. From top to bottom, concentrations of 2D CTO are 0.1 vol%, 0.06 vol% and 0.02 vol%.

Supplementary Movie 4: Description

Mechano-chromic effect of the MBhydrogel-31. First/ second half of the video display the colour evolution with the pressing force for the viewing direction to be parallel/perpendicular to the force.
